# Disqualification of Donor and Recipient Candidates From the Living Kidney Donation Program: Experience of a Single-Center in Germany

**DOI:** 10.3389/fmed.2022.904795

**Published:** 2022-06-10

**Authors:** Melissa Grigorescu, Stephan Kemmner, Ulf Schönermarck, Isidora Sajin, Wolfgang Guenther, Tiago Lemos Cerqueira, Ben Illigens, Timo Siepmann, Bruno Meiser, Markus Guba, Michael Fischereder, Manfred Johannes Stangl

**Affiliations:** ^1^Division of Nephrology, Department of Internal Medicine IV, University Hospital Munich, Ludwig-Maximilians University (LMU), Munich, Germany; ^2^German Sites Development Principles and Practice of Clinical Research Harvard T.H., Chan School of Public Health, Dresden International University, Dresden, Germany; ^3^Transplant Center, University Hospital of Munich, Ludwig-Maximilians University (LMU), Munich, Germany; ^4^Department of Kidney Transplant, Hospital Evangelico de Minas Gerais, Belo Horizonte, Brazil; ^5^Department of Neurology, University Hospital Carl Gustav Carus, Technische Universitaet Dresden, Dresden, Germany; ^6^Department of General, Visceral, and Transplant Surgery, University Hospital of Munich, Ludwig-Maximilians University (LMU), Munich, Germany

**Keywords:** living kidney donation, living donor candidates, disqualification living kidney donors, end-stage kidney disease, kidney transplantation

## Abstract

**Background:**

Kidney transplantation is the best treatment option for patients with end-stage kidney disease (ESKD) with a superiority of graft survival after living kidney donation (LKD) compared to deceased donation. However, a large part of potential donors and recipients are ineligible for LKD. Here, we analyze the leading causes for disqualification of potential living donor-recipient pairs from the LKD program and the health-related consequences for ESKD patients excluded from the LKD program in a German transplant center.

**Methods:**

In this single-center retrospective cohort study we evaluated all candidates (potential donors and recipients) presenting for assessment of LKD from 2012 to 2020 at our transplant center. Thereby we focused on candidates excluded from the LKD program. Main reasons for disqualification were categorized as medical (donor-related), psychosocial, immunological, recipient-related, and unknown.

**Results:**

Overall, 601 donor-recipient pairs were referred to our transplant center for LKD assessment during the observation time. Out of those, 326 (54.2%) discontinued the program with 52 (8.7%) dropouts and 274 (45.6%) donor-recipient pairs being ineligible for LKD. Donor-related medical contraindications were the main reason for disqualification [139 out of 274 (50.7%) potential donors] followed by recipient-related contraindications [60 out of 274 (21.9%) of potential donor-recipient pairs]. Only 77 out of 257 (29.9%) potential recipients excluded from the LKD program received a kidney transplant afterward with a median waiting time of 2 (IQR: 1.0–4.0) years. Overall, 18 (7.0%) ESKD patients initially declined for LKD died in this period.

**Conclusion:**

A large percentage of donor-recipient pairs are disqualified from the German LKD program, mostly due to medical reasons related to the donor and with partly severe consequences for the potential recipients. For these, alternative solutions that promptly enable kidney transplantation are essential for improving patient quality of life and survival.

## Introduction

Although kidney transplantation (KTx) confers the best survival benefit for patients with end-stage kidney disease (ESKD), the number of patients on the waiting list for KTx significantly exceeds the available donor kidneys worldwide (1). Living kidney donation (LKD) is one way to close this shortage with improved long-term graft and patient survival compared to KTx after deceased donation (2). Reports on global LKD rates vary widely, with countries such as Japan reporting a 90% LKD rate whereas northern-European countries attain roughly 15–30% (1, 3, 4). In Germany, LKD represents 25–30% of all donations from 2012 to 2020 with a slight decrease in the past years (1). The benefits of LKD over deceased KTx are mainly given by the overall better organ quality and the feasibility of pre-emptive transplantation as well as ABO- and human leucocyte antigen (HLA)-incompatible transplantation (5, 6). However, these recipient-related benefits should be carefully weighed against the perioperative morbidity, mortality and long-term risks for cardiovascular morbidity that potential healthy donors are exposed (6). Current guidelines for LKD evaluation providing recommendations for the transplant community show some differences in acceptable thresholds for living donors, which, among other factors, explain the variability of donor acceptance in transplant programs worldwide (7–11). These differences are evidenced by several studies reporting on the proportion and the reasons for exclusion of prospective living donors (12–14). However, data on why potential donors are disqualified for LKD in Germany are lacking. This explorative analysis evaluates the exclusion rates and the reasons for disqualification of potential donors and recipients for LKD in a transplant center in Germany. We further report the health-related consequences for ESKD patients excluded from the LKD program.

## Materials and Methods

This is a single center, retrospective cohort study concerning all potential kidney donors and respective recipients that presented for initial assessment at the LKD program of the transplant center of the LMU University Hospital in Munich from January 2012 to December 2020. The follow-up period for patients with ESKD was until December 2021. The study protocol was approved by the local ethics committee of the LMU Munich (Project number 21-0563).

### The Living Kidney Donation Evaluation Program at the Ludwig-Maximilians University Munich Hospital

**FIGURE 1:**
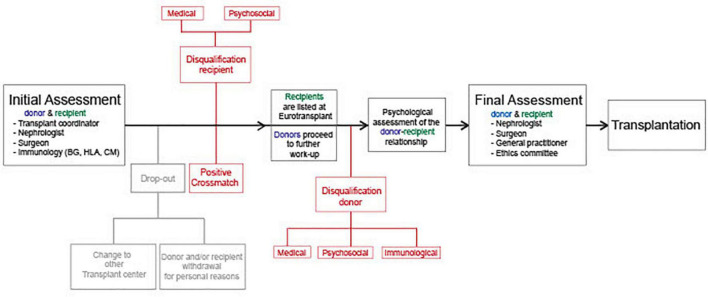
Flow chart of the LKD program: Flow chart depicting the assessment of potential living kidney donors and recipients at the LMU University Hospital Munich. The black arrow represents a timeline with the stages of the living kidney donor (LKD) program leading to successful transplantation. Donors and/or recipients withdrawing the LKD program due to relocation to another transplant center or undetermined personal reasons are highlighted in gray. Recipients and/or potential donors disqualified from the LKD program are represented in red. BG, blood group; HLA, human leucocyte antigen; CM, crossmatch.

Potential donors and recipients were evaluated according to the LKD program protocol of our institution. Figure 1 illustrates a flow chart of the LKD evaluation program. First, ESKD patients and potential donors are referred by a primary care nephrologist for the initial assessment. A team consisting of a transplant coordinator, a transplant surgeon and a nephrologist conduct the first consult. Blood samples from potential donors and recipients are obtained for immunological analysis. The immunology department reports on blood group, HLA typing, antibody detection, and crossmatch. The potential recipient is evaluated independently from the donor and, if no contraindications are yielded, the patient can be listed at the deceased donor waiting-list of the Eurotransplant kidney allocation system (ETKAS) or Eurotransplant senior program (ESP) of Eurotransplant (ET). The donor medical work-up progresses simultaneously according to recommendations of the KDIGO Guidelines. If the donor does not present contraindications, both recipient and donor undergo psychological evaluation, where the individuals and the relationship between them are examined by a psychologist. Upon completion, both donor and recipient must present for final assessment at our transplant center. Here, a nephrologist, a transplant surgeon and a general practitioner re-evaluate the findings of both candidates. Finally, assessment by an independent ethics committee of the state’s medical association is necessary. After acceptance by all the above, surgery is planned as best estimated by the medical staff, the donor and the recipient. Candidates (potential donors and recipients) withdrawing the LKD program for personal reasons or voluntarily changing the transplant center before assessment completion are categorized as drop-outs (Figure 1, highlighted in gray). All other candidates (potential donors and recipients) that yield any contraindications are highlighted in red (Figure 1).

### Disqualification Criteria and Study Population

The study population included all potential donors and recipients that presented for the first assessment of the LKD program at our transplant center. For the present analysis, donors and recipients were analyzed as couples in order of presentation (donor-recipient pairs). However, potential recipients were allowed to present with two or more donors, representing an independent donor-recipient pair. The criteria for disqualification of the potential donor-recipient pairs at the LKD program were categorized as medical (donor-related), immunological, psychosocial, recipient-related and unknown. The latter includes all donor-recipient pairs excluded from LKD where reasons for disqualification were not documented. Absolute and relative contraindications for potential donors assessed for LKD are listed in Table 1. It is worth mentioning that potential donors with an initially estimated glomerular filtration rate (eGFR) and a calculated creatinine clearance by 24-h collection urine around the threshold of acceptance were subsequently referred to renal nuclear scan (specifically Technetium-99m-diethylene-triamine-pentaacetate (Tc-99m-DTPA) scan) for further evaluation. Therefore, disqualified donors due to impaired kidney function were finally excluded based on measured GFR in Tc-99m-DTPA scans (see Table 1). Potential donors with relative contraindications were analyzed in a case-dependent manner depending on the individual risk (Table 1). Absolute and relative contraindications were based on KDIGO Guidelines and adjusted to the current version of the manual for evaluation of kidney transplant candidates by the working group of kidney transplant centers in North Rhine-Westphalia (15). Of note, ABO- and HLA-incompatibility were not considered absolute contraindications, contrary to previous published data (Table 1) (16). This is due to meanwhile established treatment methods that enable ABO- and HLA-incompatible transplantations (17). ABO-incompatible transplantations were analyzed case dependently. No IgG/IgM isoagglutinin-titer threshold was defined as exclusion criteria; however, preoperative desensitization was mandatory. Also, HLA-incompatible transplantations were analyzed in a case dependent manner. Recipients with a high titer of donor-specific antibodies (DSA) (i.e., mean fluorescent intensity (MFI) > 10,000 as well as a positive B- and T-cell cross-match were excluded. Patients with either Luminex-detected DSA with an MFI > 3,000 and a negative cross-match or, a positive CDC-B-cell and/or Luminex cross-match and MFI < 3,000 were accepted after individual case discussion. Pre-operative desensitization was mandatory if accepted for LKD.

**TABLE 1:** Absolute and relative contraindications of potential donors for LKD.

Absolute
Medical
Age < 18 years old
Impaired kidney function^#^
mGFR < 70 ml/min 1.73 m^2^
Nephrological
Manifest kidney disease (e.g., Alport syndrome)
Glomerular microhematuria (with signs of kidney disease)
Proteinuria and/or Albuminuria (>300 mg/d)
Cardiovascular
Hypertension [poorly controlled (>140/90 mmHg) with more than two medications]
Diabetes (any type) or pathological oGTT
Active smoking
Arteriosclerosis (as assessed by Doppler ultrasound or CT scan)^§^
BMI > 35 kg/m^2^ (without weight loss)
Urological
Incidental abnormal kidney cysts, vessels or ureter
Unclear incidental macrohematuria
Nephrolithiasis or high risk for nephrolithiasis
Malignancy
Active (excluding treatable *in situ* carcinoma such as prostate cancer Gleason < 6
Non-melanoma skin cancer, *in situ* bladder-carcinoma, *in situ* cervical cancer)
In recent past medical history
Active infectious disease (Hepatitis B/C, HIV, TBC)
Genetic disorders associated with kidney disease (e.g., polycystic kidney disease)
Psychiatric disease
Immunological*
Positive crossmatch
Psychosocial
No meaningful relationship between donor and recipient*
Signs of coercion*
Uncertainty for transplantation
Active substance abuse (alcohol, illicit drugs)
**Relative**
Medical
Age (18–35 years old)
Case-dependent
Immunological*
HLA Antibodies
Blood group incompatibility
Psychosocial
Case-dependent

LKD, living kidney donation. *Donor- and recipient related contraindication. ^#^As assessed by renal nuclear scan. ^§^Risk assessment by the radiologist and transplant surgeon. mGFR, measured glomerular filtration rate; BMI, body mass index; oGTT, oral glucose tolerance test; HIV, human immunodeficiency virus; HLA, human leucocyte antigen; TBC, tuberculosis.

Recipient-related contraindications included any relevant medical or psychological conditions attaining a higher risk for the recipient. Table 2 shows the most relevant absolute and relative medical and psychological conditions that exclude potential recipients from the LKD program based on KDIGO Guidelines (18). Patients with multiple comorbidities were recipients with at least three advanced medical conditions, among them at least one or the combination of them implying a significant reduction of the patients’ estimated survival according to the standards in Germany (Table 2). Under relative contraindications we include conditions which can be changed or resolved over time, therefore only delaying LKD assessment, and/or conditions that should be assessed individually. Here, a too long dialysis vintage (i.e., over 8 years) and thus a period of time resembling the average waiting time for ESKD patients on the deceased kidney transplant list in Germany with a reasonable chance of receiving a deceased kidney in a short period of time, and a stable kidney function, defined by an eGFR of at least 15 ml/min and a low likelihood for progression of ESKD in need for renal replacement therapy for the next 6 months, were included. In many cases, potential donors and recipients presented with more than one contraindication for LKD. Donor-recipient pairs presenting with more than one relative contraindication were evaluated in a multidisciplinary team as mentioned above.

**TABLE 2:** Absolute and relative contraindications for potential recipients for LKD.

Absolute
Medical
Cardiovascular*
Severe cardiac disease with uncorrectable symptoms (NYHA III/IV),
ventricular dysfunction (ejection fraction < 30%), severe valvular disease)
Pulmonary*
Severe irreversible obstructive or restrictive disease
Gastroenterological*
Acute decompensated liver cirrhosis**
Malignancy*
Active (except *in situ*/low grade carcinoma: e.g., prostate cancer with Gleason score < 6 or
incidental detected renal tumors < 1 cm max diameter)
In recent past medical history (only low-grade tumor at least 2 years low grade tumor without recurrence)
Multiple comorbidities^§^
Neurological*
Progressive central neurodegenerative disease
Unstable psychiatric disorder*
Psychosocial
No meaningful relationship between donor and recipient
Coercion
Non-adherence
Uncertainty for transplantation
**Relative**
Medical
BMI > 35 kg/m^2^ (without weight loss)
Cardiovascular*
Active, symptomatic cardiac disease (unassessed)
Active, symptomatic peripheral arterial disease
Neurological*
Recent stroke or transient ischemic attack
Gastroenterological*
Active disease (e.g., peptic ulcers, acute pancreatitis, infections, uncontrolled inflammatory bowel disease, acute hepatitis)
Endocrinological*
Severe hyperparathyroidism (PTH > 800 pg/ml under conservative therapy and unsuitable for surgery)
Infectious disease (urinary tract infection, Anti-HCV positive)
Long dialysis vintage (over 8 years)
Stable kidney function (eGFR > 15 ml/min without worsening to RRT in 6 months)

LKD, living kidney donation. *In all categories, the statement of an expert in the field (e.g., cardiologist, pulmonologist, oncologist) was included in the evaluation process. **Consider simultaneous liver-kidney transplantation. ^§^Patients with at least three medical conditions in which at least one of them or de combination leads to a significant reduction of the patients’ survival as of Germany’s current standards. NYHA, New York, Heart Association (classification of symptomatic heart failure); BMI, body mass index; HIV, human immunodeficiency virus; PTH, parathormone; HCV, hepatitis C; eGFR, estimated glomerular filtration rate.

### Data Acquisition, Statistical Analysis and Endpoints

All data was collected between August and December 2021 from patient files and the hospital information system (KAS and LAMP, SAP) in the transplant center or from the donor and recipient data in the Eurotransplant Network Information System (ENIS). Statistical analyses were performed using Microsoft Excel version Microsoft Office 365 (Microsoft Corporation, Redmond, Washington, U.S.), and GraphPad Prism version 7.05 (GraphPad Software, LLC, San Diego, California, United States). Continuous variables were assessed for normality using histograms and Shapiro-Wilk test. Measures of central tendency and dispersion were expressed as mean and standard deviation for normally distributed data, and median and interquartile range for non-normally distributed data. Categorical variables are expressed as number of cases and percentage of total (%). For comparing continuous variables student’s *t*-test and Mann-Whitney-*U*-test were used for normally and non-normally distributed data, respectively. Categorical variables were compared using Fisher’s exact test or Pearson’s Chi-square test. A *p*-value < 0.05 was considered as statistically significant. Missing data from the LKD program assessment were assumed as missing completely at random (MCAR). Missing data from recipient follow-up were assumed as missing not at random (MNAR). The primary outcome includes the rate and the summary of reasons for disqualification of potential living kidney donors and recipients. The secondary outcome is the impact on the potential recipients in respect to transplantation and mortality.

## Results

**FIGURE 2:**
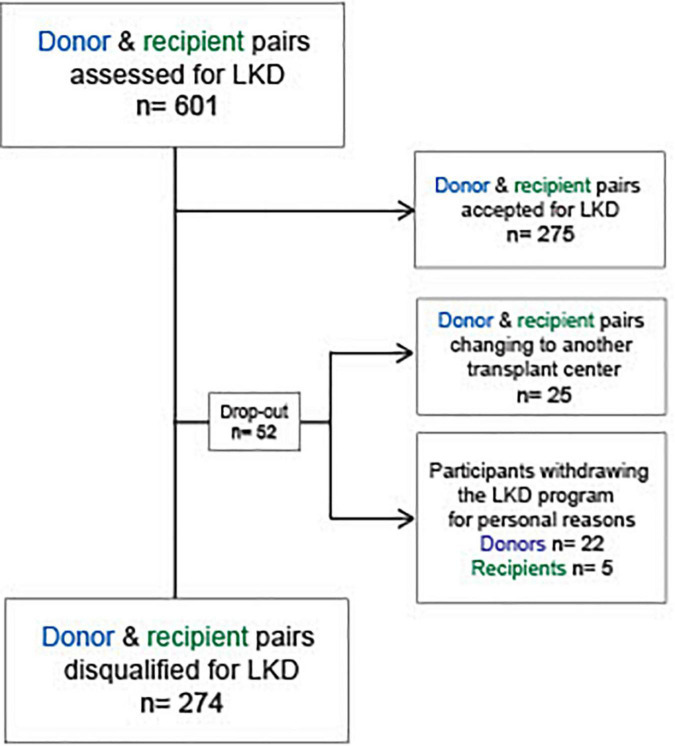
Flow diagram: Study design. LKD, living kidney donation; n, number.

Between 2012 and 2020, 601 potential living kidney donor-recipient pairs presented for initial assessment at the transplant center of our institution. 275 (45.8%) proceeded for living kidney donation after successfully completing the LKD program. In total, 326 (54.2%) potential donor-recipient pairs did not complete the LKD program. Out of these, 52 (8.7%) accounted for drop-outs with 25 (4.2%) prospective donor-recipient pairs relocating to another transplant center and other 27 (4.5%) (22 potential donors and 5 potential recipients) withdrawing from the program for personal reasons. Overall, 274 (45.6%) potential donor-recipient pairs were disqualified for LKD. The study flow diagram is depicted in Figure 2. Among all evaluated candidates (accepted and declined donor-recipient pairs), the proportion of men as potential recipients (independent of the evaluation outcome) was higher than of women (340 vs. 192, respectively) (Supplementary Table 2). Accordingly, women presented more frequently as potential donors (independent of the evaluation outcome) than men (314 vs. 235, respectively, *p* ≤ 0.0001) (Supplementary Table 2).

**FIGURE 3:**
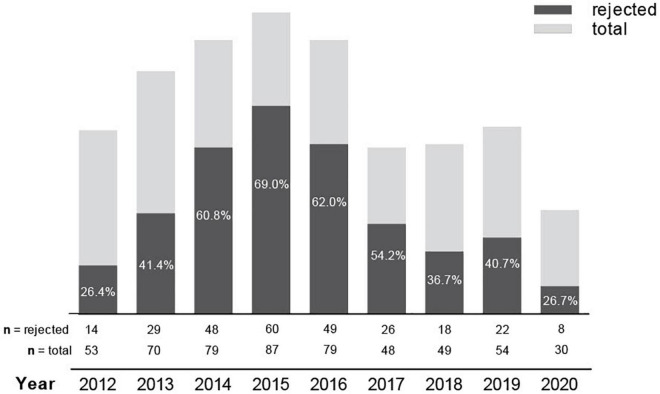
Percentage of potential donors disqualified for LKD per year from 2012 to 2020:%, percentage; n, number.

The proportion of potential donors-recipient pairs excluded for LKD between 2012 and 2020 per year at our transplant center is depicted in Figure 3. The graphic shows the highest disqualification rates in the years 2014–2016 with over 60% of potential donor-recipient pairs being ineligible for LKD. During that period, the absolute number of potential donor-recipient pairs evaluated for LKD was also higher and, compared to other years, potential recipients presented more frequently with two or more donors for the initial LKD evaluation. From 2017 until 2020, a marked reduction in disqualification rates and absolute number of evaluated donor-recipient pairs was observed. However, the overall number of donor-recipient pairs accepted for LKD per year remained similar during the evaluation period (Figure 3).

### General Characteristics of Potential Donors and Relationship to Respective Recipients

General characteristics of potential donors declined for LKD and donors accepted for LKD are shown in Table 3. Median age at presentation was 55.5 (IQR: 48.0–63.0) and 56.0 (IQR: 49.0–61.0) years in disqualified and accepted donors, respectively, without a statistical difference between groups (*p* = 0.82). There was overall a higher proportion of women presenting as potential donors (56.6 and 57.8% in disqualified donors and accepted donors, respectively, *p* = 0.79). Conversely, the donor-recipient relationship differed significantly between the groups with parents (45.1%) showing the highest rate among accepted donors, and spouses (37.8%) the highest rate among disqualified potential donors (*p* = 0.0017). No acquaintances were accepted as donors for LKD (see Table 3).

**TABLE 3:** General characteristics of disqualified donors and donors completing the LKD program.

Characteristics	Disqualified donors *n* = 274	Accepted donors *n* = 275	*p*-value
Age in years in median (IQR)	55.5 (48.0–63.0)	56.0 (49.0–61.0)	0.82
Range	25–87	29–80	
Gender, *n* (%)			
Male	119 (43.4)	116 (42.2)	0.79
Female	155 (56.6)	159 (57.8)	
Relationship to recipient, *n* (%)			
Parents	80 (29.2)	124 (45.1)	0.0017
Spouse or partner	105 (38.3)	91 (33.1)	
Sibling	39 (14.2)	35 (12.7)	
Second degree relative	17 (6.2)	8 (2.9)	
Friend	18 (6.6)	9 (3.3)	
Other relatives*	10 (3.6)	8 (2.9)	
Acquaintance	4 (1.5)	0 (0.0)	

Comparison of groups by Fisher’s exact test or Pearson’s Chi-square test for categorical data and Mann-Whitney U-test for continuous non-parametric data. *Includes stepfather, father-in-law, mother-in-law, brother-in-law, sister-in-law. n, absolute number; IQR, interquartile range; %, percentage.

### Reasons for Disqualification of Potential Living Kidney Donor-Recipient Pairs

**FIGURE 4:**
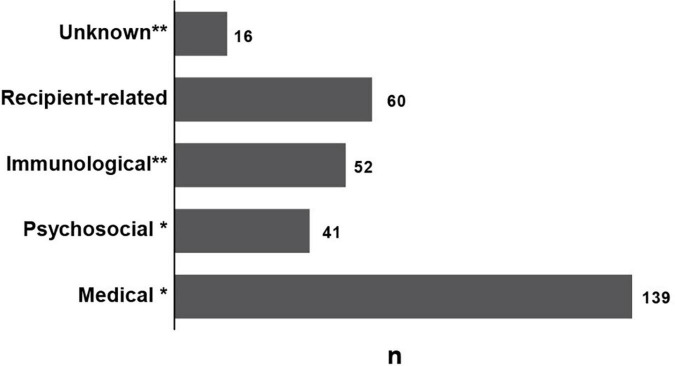
Reasons for disqualification of the potential donor-recipient pairs from the LKD program: n: number. *Donor-related contraindications. **Donor- and/or recipient-related contraindications.

In the 9-year period, 274 (45.6%) potential donor-recipient pairs were ineligible for living kidney transplantation. The reasons for disqualification of the donor-recipient pairs are depicted in Figure 4. Half of the potential donor-recipient pairs [139 (50.7%) out of 274] were ineligible due to medical reasons related to the donor. Recipient-related issues were the second highest cause for exclusion with 60 (21.9%) cases, followed by immunological and psychosocial issues related to the donor [52 (18.9%) and 41 (14.9%) out of 274 cases, respectively]. In 16 (5.8%) cases, no specific reason for exclusion was documented (Figure 4, denoted as unknown). Only in 3 cases potential donors were excluded due to the presence of an alternative, more suitable candidate. It is worth mentioning that some of the disqualified donor-recipient pairs exhibited two or more reasons for disqualification. In one case, a potential donor was diagnosed with an esophageal submucosal mass, delaying the work-up due to its clarification. Meanwhile, profound non-adherence of the potential recipient was documented. Consequently, this donor-recipient pair was disqualified from the LKD program upon interdisciplinary decision. Another notable example shows a potential recipient with a low titer of donor specific HLA antibodies, considered a relative contraindication. However, the potential recipient yielded psychological issues in the following work-up, excluding the donor-recipient pair from the program.

The leading cause for exclusion due to medical reasons among donors (139 of potential donors) was reduced kidney function in 42 (30.2%) cases, followed by cardiovascular risk factors including a body mass index (BMI) over 35 kg/m^2^ in 23 (16.5%) cases without weight loss in the follow-up examination and poorly controlled hypertension in 17 (12.9%) cases (Table 4). Remarkably, 15.1% (21 out of 139 potential donors with medical contraindications) were diagnosed with a malignant disease during work-up, with prostate cancer representing one third of the newly diagnosed malignancies (7 out of 21 cases), followed by renal cell carcinoma (4 out of 21 cases) (Supplementary Table 1). All patients with incidental prostate cancer had a Gleason score of at least 7. Patients with adequate treatment and at least a 2-year recurrence-free period were reconsidered for LKD. Further incidental malignant diseases are listed in Supplementary Table 1. Overall, more men were newly diagnosed with a malignant disease [13 (61.9%) out of 21 potential donors]. The mean age of potential donors with incidental malignant disease was 63.8 ± 9.3 years and 65.1 ± 12.1 years in donors with incidental prostate cancer (Supplementary Table 1). Other relevant medical exclusion criteria involved nephrological issues [14 (10.1%) out of 139 potential donors with medical contraindications] with incidental diagnosis of proteinuria or manifest kidney disease (Table 4). For example, one potential donor was diagnosed with Alport syndrome and another with hypertensive nephropathy. Furthermore, three blood-related donors were excluded due to genetical abnormalities that increased the risk for kidney disease of the donor. Two potential donors presented genetical variants leading to focal segmental glomerulosclerosis and one potential donor had a genetical variant that increased the risk for developing atypical hemolytic uremic syndrome. It is worth mentioning that also among donors excluded for medical reasons, 44 yielded two or more absolute and/or relative exclusion criteria.

**TABLE 4:** Donor-related reasons for disqualification from the LKD program.

Medical *n* (%)	*n* = 139
mGFR < 70 mL/min/1.73 m^2#^	42 (30.2)
Nephrological*	14 (10.1)
Urological**	12 (8.6)
Cardiovascular risk factors	
Hypertension (poorly controlled with more than two medications)	17 (12.9)
Diabetes or pathological oGTT	14 (10.1)
Smoking	9 (6.5)
Arteriosclerosis	6 (4.3)
BMI > 35 kg/m^2^ (without weight loss in the work-up)	23 (16.5)
Age (too young)	7 (5.0)
Malignancy	28 (20.1)
In recent past medical history	7 (5.0)
Diagnosed during work-up	21 (15.1)
Psychiatric	7 (5.0)
Lung disease	4 (2.9)
Genetical predisposition for kidney disease	3 (2.2)
Active infectious disease (Hepatitis B/C, TBC, or HIV)	4 (2.9)
Other***	5 (3.6)
**Psychosocial** *n* (%)	***n* = 41**
Psychological assessment	10 (24.3)
Insufficient bond between donor and recipient	5 (12.2)
Other social aspects****	15 (36.5)
Uncertainty for transplantation	5 (12.2)
Signs of coercion	2 (4.9)
Financial problems	6 (14.6)
Non-adherence	4 (9.8)
Religion	2 (4.9)
**Immunological**^§^ *n* (%)	***n* = 52**
Positive crossmatch	21 (40.4)
Donor specific HLA Antibodies	31 (59.6)

^#^As assessed by renal nuclear scan. *Includes incidental unclear microhematuria and/or proteinuria, newly diagnosed kidney disease (e.g., Alport syndrome). **Includes incidental abnormal kidney cysts, abnormal kidney vessels or ureter, unclear incidental macrohematuria and/or nephrolithiasis or high risk for nephrolithiasis. ***Includes neurological abnormalities (newly diagnosed multiple sclerosis), one case of Merkelsson-Rosenthal Syndrome, and gastrointestinal abnormalities. ****Includes difficult social circumstances such as single parents of small children, planning child conception. Some candidates qualified for more than one category. n, number; mGFR, measured glomerular filtration rate; oGTT, oral glucose tolerance test; HIV, Human immunodeficiency virus; HLA, human leucocyte antigen; TBC, tuberculosis.

*^§^donor-related contraindications depending on the potential recipient.*

Overall, 52 (19.0%) out of the 274 potential donor-recipient pairs assessed were declined due to immunological reasons. 21 (40.4%) out of 52 cases had a positive crossmatch. In the remaining 31 (59.6%) out of 52 cases, donor specific HLA antibodies were detected and yielded an increased immunological risk, accounting for a relative contraindication. Immunological contraindications were more frequent in female recipients than in men [29 (55.8%) vs. 23 (44.2%) of potential recipients, respectively]. No donor-recipient pairs were excluded due to ABO-incompatibility with some of the participants undergoing ABO-incompatible transplantation upon desensitization of the recipient. However, in some cases an alternative ABO-compatible candidate was considered as more suitable for LKD.

Relevant psychosocial reasons for exclusion of the donor represented 14.9% (41 out of 274 declined potential donors). Ten (23.8%) out of 41 potential donors were declined due to psychological assessment, mostly due to insufficient bond between the potential donor and recipient (Table 4). Uncertainty for transplantation was also a frequent cause for exclusion with 12.2%. Other social aspects (15 out of 42 cases) leading to exclusion of the donor included complex social circumstances such as being a single parent of small children or conflicts between the potential donor and recipient. Less common reasons in our cohort were signs of coercion, financial problems, signs of non-adherence and religion-related reasons.

### General Characteristics of Recipients of Disqualified Donor-Recipient Pairs and Recipients Who Underwent Living Kidney Transplantation

The following section focuses on all potential recipients declined for LKD, independent of the reason (donor- or recipient-related). Out of the 326 potential donor-recipient pairs who did not conclude the LKD program, 32 recipients presented with two or more potential donors, leading to a total of 257 potential recipients disqualified from the program in this time period (after excluding donor-recipient pairs relocating to another transplant center and recipient drop-outs). Table 5 shows the general characteristics of recipients disqualified for LKD and recipients accepted for LKD. Patients who underwent living donation were significantly younger than recipients disqualified from the LKD program [44 (29.0–55.0) years and 49 (36.5–58.0) years, respectively, *p* = 0.0007]. The proportion of men as potential recipients for LKD was higher in both groups (185 (67.3%) successfully transplanted recipients and 155 (60.3%) recipients of disqualified donor-recipient pairs) with no significant difference between accepted and declined recipients (*p* = 0.104). The rate of pre-emptive evaluated recipients with a successful LKD and recipients disqualified for LKD was not different (33.1 and 28.0%, respectively, *p* = 0.22). Also, no significant difference was found in respect to the proportion of patients with a previous kidney transplant between successfully transplanted recipients and recipients from disqualified donor-recipient pairs [39 (14.2%) vs. 46 (17.9%), respectively, *p* = 0.29] (Table 5). Finally, the median dialysis vintage of ESKD patients accepted for LKD was 0.75 (IQR: 0.75–1.75) years until successfully performed kidney transplantation.

**TABLE 5:** Baseline characteristics of recipients from disqualified donors and recipients who underwent LKD.

General characteristics	Recipients disqualified for LKD* *n* = 257	Recipients who underwent LKD *n* = 275	*p*-value
Age in years (median, IQR)	49 (36.5–58.0)	44 (29.0–55.0)	0.0007
Range	2–80	1–77	
Gender, *n* (%)			
Male	155 (60.3)	185 (67.3)	0.104
Female	102 (39.7)	90 (32.8)	
Preemptive transplantation, *n* (%)	72 (28.0)	91 (33.1)	0.22
Previous kidney transplant, *n* (%)	46 (17.9)	39 (14.2)	0.29

Comparison of groups by Fisher’s exact test or Pearson’s Chi-square test for categorical data and Mann-Whitney U-test for continuous non-parametric data. n, number; %, percent; IQR, interquartile range. *Independent on the reason for disqualification (donor- or recipient-related).

### Recipient-Related Reasons for Disqualification and Outcomes of Recipients Disqualified From the Living Kidney Donation Program

We report that 60 (21.9%) out of 274 potential donor-recipient pairs were ineligible for LKD due to recipient-related issues. Median age in this group was 50 (41.8–63.8) years. There was no significant difference in terms of gender within this group [35 (58.3%) men vs. 25 (41.7%) women declined, *p* = 0.76]. Table 6 displays the medical and psychosocial reasons for exclusion of potential recipients from the LKD program. In most of the cases, recipients were declined due to medical reasons. Multiple comorbidities and acceptable or improved kidney function of the potential recipients were among the leading causes for exclusion [9 (15.0%) and 11 (18.3%), respectively]. Also in this group, incident malignant disease represented an important exclusion criterion with 11 (18.3%) out of 60 cases disqualified (Table 6). More men were diagnosed with incidental malignant disease among potential recipients (6 (75%) out of 8 potential recipients) (Supplementary Table 1). Three of them were diagnosed with prostate cancer. Cardiovascular complications as well as long dialysis vintage were found in 5 (8.1%) cases, respectively. Four (6.3%) patients received a deceased kidney during the work-up or changed to the ESP program, while other four (6.3%) were listed for kidney-pancreas transplantation, due to better outcomes. Three patients (4.8%) died during the work up.

**TABLE 6:** Recipient-related reasons for disqualification from LKD program.

Recipient-related contraindications	*n* = 60
**Medical** *n* (%)	
Multiple comorbidities	9 (15.0)
BMI > 35 kg/m^2^ (without weight loss during evaluation)	3 (5.0)
Malignancy	11 (18.3)
Prostate cancer	3 (5.0)
Other malignancies*	8 (13.3)
Cardiovascular complications	5 (8.3)
Death during LKD evaluation	3 (5.0)
Long dialysis vintage	5 (8.3)
Stable kidney function	11 (18.3)
Received deceased kidney or changed to ESP program	4 (6.7)
Listed for simultaneous kidney-pancreas transplantation	4 (6.7)
Other**	9 (15.0)
**Psychosocial** n (%)	
Psychological assessment	4 (6.7)
Non-adherence	1 (1.7)
Other***	2 (3.3)

*Includes Non-Hodgkin lymphoma, leukemia, melanoma. **Includes uncontrolled hyperparathyroidism, multiple abscesses, chronic pancreatitis. ***Includes insecurity and anxiety of the recipient toward LKD. Some patients qualified for more than one category. n, number; %, percent.

Overall, 78.9% (203 out of 257) of potential recipients initially declined from the LKD program remained in contact with our transplant center. Following disqualification, 77 (29.9%) ESKD patients received a kidney transplant and almost half of those (48.1%) received a kidney from an alternative living donor. The median time to KTx was overall 2 (IQR: 1.0–4.0) years. The latter was significantly shorter for recipients of living kidney donors than for recipients of deceased donors (1 (0–2) year vs. 4 (1.5–5.0) years, respectively, *p* = 0.0001). 18 (7.0%) out of 257 potential recipients initially declined at the LKD program died within the follow-up period, with only three of them receiving a deceased kidney transplant after exclusion from the LKD program. Unfortunately, we have no information regarding transplantation or death rate of 54 (21.1%) out of all potential recipients initially declined at our LKD program.

## Discussion

In Germany, only 20–30% of kidney transplants are from living donors in spite of its clear benefit for ESKD patients compared to deceased KTx (2). High disqualification rates of potential donors upon evaluation account for this problem. Nevertheless, thorough screening and clinical assessment of potential healthy living donors remains indispensable to avoid any potential harm upon transplantation. Early published data show LKD is safe for living kidney donors. However, recent reports do highlight a low but significant increase in cardiovascular and ESKD risk for patients after donor nephrectomy (15, 17). This prompts healthcare professionals to be more restrictive toward acceptance of potential donors, leading to high rates of exclusion (14, 18). Additionally, differences in guidelines for the assessment of LKD have led to variations in the acceptance of potential donors among transplant centers worldwide (19). Thus, the aim of this study was to analyze the rates of exclusion of potential donor-recipient pairs in a transplant center in Germany with a thorough description of the causes and possible consequences for waitlisted patients with ESKD.

We found that 45.5% of donor-recipient pairs at our transplant center were ineligible for LKD and further 8.6% dropped-out from the program. Interestingly, the rate of potential donor-recipient pairs disqualified for LKD per year peaked between 2014 and 2016, with more recipients presenting with two or more potential donors to the initial assessment. Especially percentages of potential donors being declined for immunological and medical reasons were higher during those years. As both cross-match examinations and medical screening can also be performed by referring nephrologists prior to donor evaluation at our center, we feel the discrepancy reflects donor selection by referring nephrologists prior to presentation to our center. Additionally, data from the “Deutsche Stiftung Organtransplantation” (DSO) has revealed a marked variability in the rate of LKD, deceased kidney transplantations, and waitlisted ESKD patients in Germany over the past 20 years (19, 20). It is possible that due to the short period of time used for our analysis (9 years), such inherent variations were only insufficiently detected. Moreover, the substantial reduction in 2020 might have been a consequence of the surging global coronavirus disease 2019 pandemic.

Similar to other studies, we report that almost half of the donor-recipient pairs evaluated for LKD at our transplant center are disqualified (21–23). In 50.7% of the cases donor-related medical contraindications were the reason for exclusion with reduced mGFR and cardiovascular risk factors (obesity, hypertension and diabetes) as leading causes. Villafuerte-Ledesma et al. and Lapasia et al. report similar results (22, 23). By contrast, a study from Ireland reported different results with reduced eGFR and diabetes not playing a significant role in disqualification rates (12). This was also observed by Perlis et al., where urological pathologies prevailed as cause for disqualification of potential donors (24). However, these results should be interpreted with caution, as the structure of the LKD evaluation programs of each center differs considerably and, in the latter, patients with absolute contraindications (such as reduced eGFR) had already been excluded in a preliminary screening process. We believe that such differences among transplant centers worldwide are partly responsible for the varied disqualification rates and should be considered by clinicians when evaluating donor and recipient candidates for LKD.

One interesting aspect of our study is the high incidence of malignant disease among potential living donors with one third of the cases presenting incidental prostate cancer. These observations are probably related to the age of this group of potential donors (mean age: 65.1 ± 12.1 years), which resembles the worldwide mean age of diagnosis of prostate cancer at 66 years (25). No other studies report these findings. Unlike our results, several studies report an overall younger population presenting as potential donors with a mean age ranging between 40 and 45 years (12, 22, 26, 27). Only Gregorini et al. and Villafuerte-Ledesma et al. reported a comparable mean donor age between 53 and 55 years old (21, 23). Furthermore, our data show no difference in respect to the age of accepted and declined donors, suggesting that at our transplant center older age *per se* is not linked to donor-disqualification. On the contrary, in Spain and in Ireland older donors were more likely to be excluded from LKD (12, 23).

Corresponding to other transplant centers worldwide, we observed substantial gender differences among potential recipients and donors for LKD. Women presented overall more frequently as potential donors, independent of the evaluation outcome, which is analogous to previous published data (22, 28). Altruism and a more paternalistic approach of women toward their relatives have been associated with this finding (29). The higher proportion of men in need of a kidney transplant has been documented in other studies as well (12, 23), which has been associated to a higher risk of progression of chronic kidney disease (CKD) and ESKD among men (30). In addition, women waitlisted for a kidney transplant (especially deceased KTx) have often increased levels of preformed antibodies, reducing the likelihood of a successful transplantation (31). Nevertheless, additional factors such as socioeconomical and cultural issues should be addressed in future studies as alternative explanations for the gender disparity and potentially reduce the gap (30).

In this study, potential recipients accepted for LKD were substantially younger than potential recipients disqualified for LKD. Similar data has been reported by the DSO, where the percentage of ESKD patients between 16 and 55 years receiving a living donation was higher than in patients of the same age group receiving a deceased kidney donation (20). Reasons for this discrepancy might be related to the cause for ESKD, comorbidities in the ESKD older population and timing of patient referral by the primary care nephrologist. This trend highlights that kidney transplantation in the increasingly older ESKD population in Germany is mostly dependent on deceased kidney donation, reducing their probability for receiving a kidney transplant due to the longer waiting times. Therefore, timely evaluation of recipient candidates should be pursued by treating physicians in order to make kidney transplantation an available treatment for this population.

The second most common cause for disqualification of donor-recipient pairs for LKD were recipient-related contraindications accounting for 21.9%, similar to the numbers presented by German registries in 2020 where a third of the waitlisted ESKD patients were reported unsuitable for kidney transplantation (20). Overall, medical contraindications were the most common cause for recipient disqualification from LKD. However, stable kidney function was also seen in 18.3% of the cases, reflecting the timely presentation of potential recipients and potential donors for assessment at out transplant center and the improvement of therapies for patients with CKD. One seldomly reported cause for disqualification in other centers was a too long dialysis vintage. At our transplant center, this was weighed in patients with a dialysis vintage that resembled the average waiting times for receiving a deceased kidney transplant in Germany, whereby the benefit of LKD compared to deceased kidney donation is mostly lost.

Immunological contraindications (including mostly a positive cross-match and/or presence of donor specific HLA-antibodies) accounted for disqualification of 18.9% of donor-recipient pairs assessed. ABO-incompatibility was considered a relative contraindication and no patients were excluded for this reason in our study. This is different from previous published data, where potential donors were automatically excluded upon ABO-incompatibility and this alone represented a relevant cause for disqualification ranging from 12 to 20% (16, 22, 23, 32). New therapeutic strategies have allowed for prior desensitization of recipient candidates, enabling LKD under these conditions and thus reducing disqualification rates significantly (17). However, not all patients qualify for this therapeutic approach. Careful weighing of risk and benefits and assessment by an interdisciplinary team of experts remains indispensable. Otherwise, presence of donor specific antibodies was a relevant relative contraindication present in about half of patients with immunological contraindications, implying that HLA-incompatibility still signifies a higher risk for clinicians. Nevertheless, ABO-incompatible transplantations and HLA-desensitization have shown promising results with comparable graft and patient survival and should be available for all candidates assessed for LKD (17).

We report that almost 15% of potential donors presented psychosocial contraindications for LKD, a rather higher proportion compared to other studies (14, 33). We show there is a wide range of reasons in this regard, including an insufficient bond between donor and recipient, uncertainty for transplantation, non-adherence and legal issues as signs of coercion. Psychosocial factors should not be underestimated regarding KTx and LKD, especially in Germany where the number of LKD has shown a progressive decline, in part due to more stringent criteria regarding psychosocial factors for donor selection (34). One group from the United Kingdom proved that socioeconomical, geographical and demographic factors are strongly associated with the likelihood of receiving a LKD compared to clinical factors (35). Disqualification due to uncertainty for transplantation remains a relevant issue and highlights the need for a better education of potential donors and recipients regarding perioperative risks and long-term consequences after donor-nephrectomy.

The long waiting times for deceased kidney transplantation in Germany (mean waiting time 8 years as of 2022) remain an important issue and are substantially longer compared to other countries (1, 36). In our study, only one third of recipient candidates initially disqualified from the LKD program obtained a kidney transplant in the following period, with about half of them receiving a deceased KTx. In addition, the median waiting time for potential recipients with deceased KTx was considerably longer, which is linked to poorer graft survival and patient prognosis. Up to 7.0% of patients died within the observation period, highlighting the severe health-related consequences waitlisted patients are subject to, in part due to the long waiting times in Germany. Only a few European countries, among them Germany, use the opt-in or informed consent system for acquiring deceased organ donors, which markedly reduces the number of available donors. Our observations clearly emphasize the need for implementing further strategies to increase the number of donor candidates, including living kidney donors.

Taken together, our study underlines the importance of a thorough clinical evaluation of potential donors and recipients for LKD, validating previous data from around the world. Further strategies, such as risk-stratification scores [e.g., living kidney donor profile index (LKDPI)], among others, should support clinicians in the decision-making process in order to provide patients with the best treatment modality (37). Furthermore, German society should evaluate the possibility of expanding the living donor pool by allowing paired exchanges or cross-over LKD and pooled donation. LKD has proven to be not only better for patient survival but also to be more cost-effective than other ESKD treatment modalities (38).

This study has some limitations. The observational, retrospective design limits the completeness of data. Additionally, data was analyzed in a period of time where changes in guidelines and clinical practice might have influenced disqualification rates. This is a single-center study and differences to other transplant centers in Germany might be considerable, therefore limiting generalizability. Nevertheless, our study is the first analysis from a German center providing information on disqualification of living kidney donor-recipient pairs. The recently introduced German living donor registry (Safety of the Living Kidney Donor (SOLKID) has encouraged the development of risk stratification scores to identify the population with increased medical and psychosocial risk upon donor nephrectomy (39). Nevertheless, additional studies from other German transplant centers are necessary in order to increase the available data and therefore create better strategies for living donor assessment and management of candidates for LKD.

In conclusion, half of potential donor-recipient pairs assessed at our LKD program are not eligible for transplantation with only a third of declined potential recipients receiving an alternative organ in the following years. Further efforts are still necessary to increase the living donor pool and reduce the gap between transplanted and wait-listed patients, always protecting the living donor from any harm.

## Data Availability Statement

The original contributions presented in this study are included in the article/Supplementary Material, further inquiries can be directed to the corresponding author.

## Ethics Statement

The studies involving human participants were reviewed and approved by the Local Ethics Committee of the LMU University Munich. Written informed consent for participation was not required for this study in accordance with the national legislation and the institutional requirements.

## Author Contributions

MGr, SK, MF, and MS designed the concept of the retrospective study. MGr and TC wrote the manuscript. SK, MF, MS, US, and TS edited and revised the manuscript. MGr, IS, WG, and TC prepared clinical data for analysis. MGu, MS, SK, MF, US, BI, BM, and TS oversaw the study and critically discussed the manuscript. All authors substantially contributed to the manuscript.

## Conflict of Interest

The authors declare that the research was conducted in the absence of any commercial or financial relationships that could be construed as a potential conflict of interest.

## Publisher’s Note

All claims expressed in this article are solely those of the authors and do not necessarily represent those of their affiliated organizations, or those of the publisher, the editors and the reviewers. Any product that may be evaluated in this article, or claim that may be made by its manufacturer, is not guaranteed or endorsed by the publisher.

## Abbreviations

CKD: chronic kidney disease
DSO: Deutsche Stiftung Organtransplantation
eGFR: estimated glomerular filtration rate
ESKD: End-stage kidney disease
ESP: Eurotransplant senior program
ET: Eurotransplant
ETKAS: Eurotransplant kidney allocation system
HLA: human leucocyte antigen
IQR: Interquartile range
KDIGO: Kidney disease: Improving Global Outcomes
KTx: Kidney transplantation
LKD: Living kidney donation
LKDPI: Living Kidney Donor Profile Index
mGFR: measured glomerular filtration rate
SOLKID: Safety of the Living Kidney Donor.
