# Corrigendum: Disqualification of donor and recipient candidates from the living kidney donation program: Experience of a single-center in Germany

**DOI:** 10.3389/fmed.2022.1004413

**Published:** 2022-11-17

**Authors:** Melissa Grigorescu, Stephan Kemmner, Ulf Schönermarck, Isidora Sajin, Wolfgang Guenther, Tiago Lemos Cerqueira, Ben Illigens, Timo Siepmann, Bruno Meiser, Markus Guba, Michael Fischereder, Manfred Johannes Stangl

**Affiliations:** ^1^Division of Nephrology, Department of Internal Medicine IV, University Hospital Munich, Ludwig-Maximilians University (LMU), Munich, Germany; ^2^German Sites Development Principles and Practice of Clinical Research Harvard T.H., Chan School of Public Health, Dresden International University, Dresden, Germany; ^3^Transplant Center, University Hospital of Munich, Ludwig-Maximilians University (LMU), Munich, Germany; ^4^Department of Kidney Transplant, Hospital Evangelico de Minas Gerais, Belo Horizonte, Brazil; ^5^Department of Neurology, University Hospital Carl Gustav Carus, Technische Universitaet Dresden, Dresden, Germany; ^6^Department of General, Visceral, and Transplant Surgery, University Hospital of Munich, Ludwig-Maximilians University (LMU), Munich, Germany

**Keywords:** living kidney donation, living donor candidates, disqualification living kidney donors, end-stage kidney disease, kidney transplantation

In the published article, there was an error regarding the affiliation for “Timo Siepmann.” Instead of having affiliation 2: *German Sites Development Principles and Practice of Clinical Research Harvard T.H., Chan School of Public Health, Dresden International University, Dresden, Germany* he should have the following affiliation: *5: Department of Neurology, University Hospital Carl Gustav Carus, Technische Universitaet Dresden, Dresden, Germany*.

In the published article, an author name was incorrectly written as “Manfred Johannes Stang.” The correct spelling is “Manfred Johannes Stangl.”

The authors apologize for this error and state that this does not change the scientific conclusions of the article in any way. The original article has been updated.

## Publisher's note

All claims expressed in this article are solely those of the authors and do not necessarily represent those of their affiliated organizations, or those of the publisher, the editors and the reviewers. Any product that may be evaluated in this article, or claim that may be made by its manufacturer, is not guaranteed or endorsed by the publisher.

