# Supplementary material for: Disqualification of Donor and Recipient Candidates From the Living Kidney Donation Program: Experience of a Single-Center in Germany
Source: Front Med (Lausanne). 2022 Jun 10;9:904795. doi: 10.3389/fmed.2022.904795 (PMC9226311; doi:10.3389/fmed.2022.904795)
Supplement: Supplementary file 1 [file Data_Sheet_1.docx]

**Supplementary tables**

| **Supplementary table 1: Malignancy diagnosis** |  |  |  |
| --- | --- | --- | --- |
|  | Number | Age (years) | Sex (male) |
| **Donors** |  |  |  |
| Malignancy (total) | 28 | 63.6 ± 8.2 | 17 |
| Incidental | 21 | 63.8 ± 9.3 | 13 |
| Prostate cancer | 7 | 65.1 ± 12.1 | 7 |
| Renal cell carcinoma | 4 | 64 ± 1.6 | 3 |
| Breast cancer | 2 | 60.5 ± 13.4 | 0 |
| Lung cancer | 1 | 66* | 1 |
| Skin cancer (melanoma) | 2 | 61.5 ± 0.7 | 0 |
| Unclear mass (not in situ) ** | 2 | 66.5 ± 7.8 | 0 |
| Plasmacytoma | 1 | 48* | 1 |
| Thyroid cancer | 1 | 67* | 1 |
| Parotid tumor | 1 | 60* | 1 |
|  |  |  |  |
| **Recipients** |  |  |  |
| Malignancy (total) | 11 | 55.3 ± 15.2 | 9 |
| Incidental | 8 | 52.4 ± 15.6 | 6 |
| Prostate cancer | 3 | 61.7 ± 9.7 | 3 |
| Renal cell carcinoma | 1 | 50* | 1 |
| Skin cancer (melanoma) | 2 | 56.0 ± 9.8 | 2 |
| Breast Cancer | 1 | 57* | 0 |
| Testicular cancer | 1 | 20* | 1 |
|  |  |  |  |
| *Individual age, as n=1. **need for biopsy | | | |

|  | | |  | |  |  |
| --- | --- | --- | --- | --- | --- | --- |
| **Supplementary table 2: Accepted vs rejected donors and recipients** | | | | | | |
|  |  |  |  |  |  |  |
|  | **Donors** n | **Recipients** n | | **p-value** | | |
| **All** |  |  | |  | | |
| male | 235 | 340 | | <0.0001 | | |
| female | 314 | 192 | |  | | |
| **Accepted** |  |  | |  | | |
| male | 116 | 185 | | <0.0001 | | |
| female | 159 | 90 | |  | | |
| **Rejected** |  |  | |  | | |
| male | 119 | 155 | | 0.0001 | | |
| female | 155 | 102 | |  | | |

**Supplementary table 2**: Fisher’s exact test was used for gender comparison between donors and recipients

among accepted, declined and overall. n: number.
